# Clinical Implementation and Perioperative Outcomes of Robot‐Assisted Pelvic Exenteration for Primary Rectal Cancer: A Multicenter Japanese Experience

**DOI:** 10.1002/ags3.70296

**Published:** 2026-10-06

**Authors:** Shoma Yamamoto, Hiroyasu Kagawa, Yusuke Yatabe, Yusuke Kinugasa

**Affiliations:** ^1^ Department of Gastrointestinal Surgery Institute of Science Tokyo Tokyo Japan

**Keywords:** laparoscopic surgery, minimally invasive surgery, pelvic exenteration, primary rectal cancer, robot‐assisted surgery

## Abstract

**Aim:**

Robot‐assisted pelvic exenteration (RPE) has been introduced as a minimally invasive option for selected patients; however, its clinical implementation, patient selection, and perioperative outcomes for primary rectal cancer remain unclear. This study describes the use and outcomes of RPE in a multicenter Japanese cohort.

**Methods:**

This retrospective observational secondary analysis included patients who underwent pelvic exenteration (PE) for primary rectal cancer at 26 Japanese institutions between January 2012 and October 2022. Patient characteristics, surgical indications, temporal trends in operative approach, and perioperative outcomes were summarized descriptively. Propensity score‐matched analyses were performed as supplementary exploratory analyses.

**Results:**

Among 135 patients, 43 (32%) underwent RPE, 57 (42%) underwent laparoscopic PE, and 35 (26%) underwent open PE. In the RPE cohort, median operative time was 690 min, median blood loss was 360 mL, and 13 patients (30%) received intraoperative transfusion. One patient (2%) required conversion to open surgery, and RM0 resection was achieved in 42 (98%). Clavien‐Dindo Grade II or higher and Grade III or higher complications within 30 days occurred in 16 (37%) and seven (16%) patients, respectively. No patient underwent reoperation during hospitalization or died within 30 days. Grade III or higher morbidity between 31 and 90 days occurred in five patients (12%). Median hospital stay was 23 days.

**Conclusion:**

RPE was performed with acceptable perioperative outcomes in selected patients with primary rectal cancer. It may serve as a complementary approach at institutions with sufficient experience in PE, whereas open surgery remains essential for extensive or technically demanding disease.

## Graphical abstract

Robot‐assisted pelvic exenteration (RPE) was performed with acceptable perioperative outcomes in selected patients with primary rectal cancer. It may serve as a complementary approach at institutions with sufficient experience in PE, whereas open surgery remains essential for extensive or technically demanding disease.

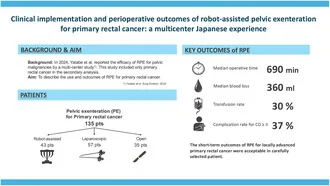

## 1 Introduction

Pelvic exenteration (PE) may represent the only opportunity for complete remission in locally advanced rectal cancer, and achieving complete resection with no microscopic residual tumor (R0) significantly improves prognosis [1, 2, 3]. With PE for primary rectal cancer, R0 resection is achievable in 80%–91% of cases [1, 2, 4, 5]. PE is a highly invasive procedure associated with high complication and mortality rates [5]. Although it is associated with deterioration in the early postoperative quality of life (QOL), most patients return to preoperative levels within 6 months [2, 6]. Improving the safety of PE while maintaining appropriate oncological indications is therefore clinically important.

Several randomized controlled trials have been conducted on minimally invasive surgery (MIS) for rectal cancer; however, they all excluded cases that required concomitant resection of other organs [7, 8, 9, 10, 11, 12]. Meanwhile, retrospective cohort studies have reported the feasibility and oncological safety of MIS for T4 rectal cancer [13, 14]. Furthermore, with the advancement of MIS, conventional laparoscopic PE (LPE) [15] and robot‐assisted PE (RPE) [16] have emerged, including procedures involving other organs.

The robot‐assisted approach has been widely adopted for malignant tumor resection because of its three‐dimensional magnified visualization, improved ergonomics, enhanced operability through tremor filtration and motion scaling, articulated instruments, and stable endoscopic platform [12, 17]. Therefore, it may also be beneficial for PE because it enables visualization with efficient and effective operation within the confined pelvic space. Nevertheless, PE encompasses heterogeneous patterns of tumor invasion and widely varying requirements for bony excision, pelvic sidewall dissection, and reconstruction. The operative approach is therefore determined not only by diagnosis but also by technical feasibility, tumor extent, institutional experience, and the multidisciplinary care framework.

A previous multicenter study by Yatabe et al., published in 2024, compared robot‐assisted, laparoscopic, and open PE for pelvic malignancies using propensity score matching [18]. For primary rectal cancer, however, the clinically relevant questions extend beyond comparative effectiveness: which patients are selected for RPE, how safely the procedure has been implemented, when open PE (OPE) remains preferable, and how RPE should be positioned within the overall PE treatment strategy. The present secondary analysis therefore aimed to describe the clinical implementation, patient selection, and perioperative outcomes of RPE for primary rectal cancer in a multicenter Japanese cohort. Comparative analyses were treated as supplementary and exploratory rather than as the primary basis for inference.

## 2 Methods

### 2.1 Study Design and Patients

This was a multicenter retrospective study including 26 institutions from the Japan Society for Endoscopic Surgery, Japan Society of Obstetrics and Gynecology, and Japan Society of Endourology and Robotics, each of which had performed at least one RPE for pelvic malignant tumors. Data were collected from all patients who underwent total PE (TPE), anterior PE (APE), or posterior PE (PPE) for radical resection of pelvic malignancies between January 2012 and October 2022. No specific inclusion criteria were established for surgeons. Patients without a histologically confirmed cancer diagnosis, those without a current cancer diagnosis, or those who refused to participate were excluded from the study. Our previous study reported the results of an initial analysis of all malignant pelvic tumors [18]. The present study included only primary rectal cancer in the secondary analysis. The principal analytic population was the full multicenter cohort, with particular emphasis on the 43 patients who underwent RPE. The three approaches were summarized descriptively to characterize their use in clinical practice; the study was not designed to establish the superiority of one approach over another.

Data regarding age, sex, height, weight, American Society of Anesthesiologists Physical Status (ASA‐PS), diagnosis, preoperative treatment, operation date, surgical approach, operative time, intraoperative blood loss, digestive and urinary reconstruction details, intraoperative complications, reoperation within 30 days, mortality within 30 days, and morbidity within 90 days were collected. The grade of each complication was determined using the CD classification [19]. The Union for International Cancer Control TNM classification (8th edition) was used for tumor staging. If necessary, data were confirmed at the main facility and queried at each facility.

### 2.2 Definitions of Surgical Technique

TPE is a procedure involving the removal of the rectum, distal colon, and genitourinary viscera, including the lower ureters and internal reproductive organs, followed by urinary reconstruction and creation of a permanent stoma. APE refers to the removal of the rectum, reproductive organs, and bladder while preserving the anus. PPE refers to the combined resection of the rectum, anus, uterus, and vagina while preserving the bladder in women. The surgical approach and procedure for each patient were decided by the surgeon.

### 2.3 Outcome Measures

Outcomes of interest included CD Grade II or higher complications, CD Grade III or higher complications, operative time, conversion, intraoperative blood loss, blood transfusion, postoperative length of stay, resection margin status, reoperation during hospitalization, 30‐day mortality, and morbidity within 90 days. RM0 was defined as no microscopic residual tumor. Clinical implementation was assessed by describing the annual number of PE procedures according to surgical approach and the timing and pattern of RPE adoption across the participating institutions.

### 2.4 Statistical Analysis

Categorical variables are presented as numbers and percentages, and continuous variables as medians with interquartile ranges. The full cohort and each surgical approach were summarized descriptively without hypothesis testing or *p‐*values in the main tables.

Supplementary exploratory propensity score–matched analyses were performed to provide clinical context among patients with overlapping measured baseline characteristics. Propensity scores were estimated using sex, neoadjuvant chemotherapy, neoadjuvant radiotherapy, PE type (TPE, APE, or PPE), and bony excision (pubectomy or sacrectomy), followed by 1:1 nearest‐neighbor matching with a caliper width of 0.2. Separate analyses compared RPE with OPE, RPE with LPE, and minimally invasive PE with OPE. Because treatment allocation was likely influenced by surgical feasibility, case complexity, institutional experience, and unmeasured anatomical factors, these analyses were not used as the primary basis for the study conclusions. Statistical analyses were performed using EZR, a graphical interface for R [20].

## 3 Results

### 3.1 Characteristics of the Full Multicenter Cohort

A total of 135 patients underwent PE for primary rectal cancer during the study period: 43 (32%) underwent RPE, 57 (42%) underwent LPE, and 35 (26%) underwent OPE (Figure 1). The median age of the entire cohort was 65 years, 119 patients (88%) were men, and the median body mass index was 22 kg/m^2^. Neoadjuvant chemotherapy and radiotherapy were administered to 87 (64%) and 55 (41%) patients, respectively.

**FIGURE 1:**
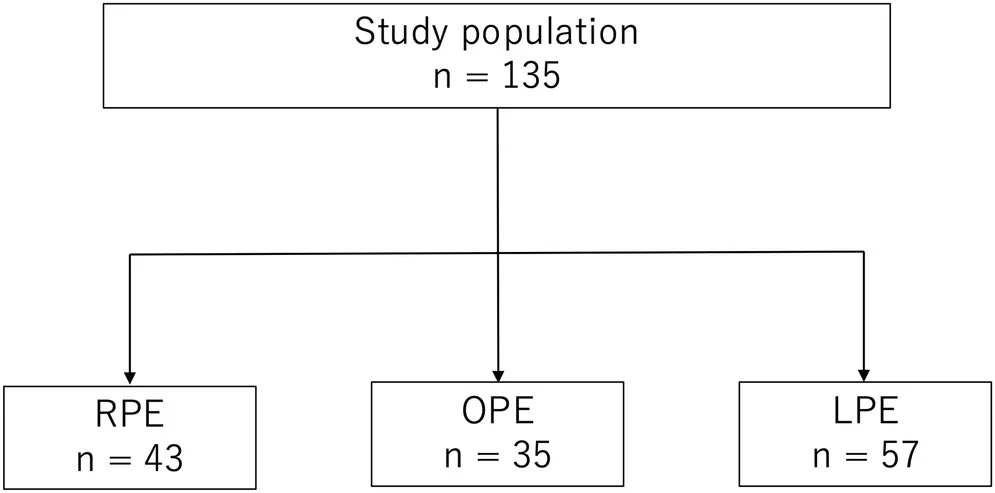
Patient selection. RPE, robot‐assisted pelvic exenteration; LPE, laparoscopic pelvic exenteration; OPE, open pelvic exenteration.

Clinical T4 disease was present in 131 patients (97%), clinically positive lymph nodes in 99 (73%), and cM1 disease at initial diagnosis in 24 (18%). Among the 24 patients with cM1 disease, 21 (88%) received neoadjuvant therapy, and four were classified as ycM0 after treatment. Patients with residual metastatic disease underwent metastasectomy concurrently with or after PE; two patients underwent simultaneous liver resection.

TPE, APE, and PPE were performed in 97 (72%), 26 (19%), and 12 (9%) patients, respectively. Bony excision was performed in six patients (4%), comprising one pubectomy and five sacrectomies. Ileal conduit urinary diversion was performed in 113 patients (84%). The baseline characteristics of the full multicenter cohort according to surgical approach are shown in Table 1.

**TABLE 1:** Baseline characteristics.

	RPE	OPE	LPE	Total
	*N* = 43	*N* = 35	*N* = 57	*N* = 135
Age	66 (57–74)	64 (58–71)	63 (52–70)	65 (56–71)
Sex				
Male	36 (84)	30 (86)	53 (93)	119 (88)
Female	7 (16)	5 (14)	4 (7)	16 (12)
BMI (kg/m^2^)	22 (19–23)	21 (19–23)	22 (20–24)	22 (19–24)
ASA‐PS				
≤ 2	33 (77)	30 (86)	55 (97)	118 (87)
≥ 3	10 (23)	5 (14)	2 (4)	17 (13)
Brinkman index				
< 400	20 (47)	23 (66)	25 (44)	68 (50)
≥ 400	23 (54)	12 (34)	32 (56)	67 (50)
Habitual alcohol consumption	18 (42)	8 (23)	14 (25)	40 (30)
History of abdominal surgery	13 (30)	15 (43)	24 (42)	52 (39)
Neoadjuvant chemotherapy	29 (67)	21 (60)	37 (65)	87 (64)
Neoadjuvant radiotherapy	13 (30)	12 (34)	30 (53)	55 (41)
Clinical tumor category (primary)				
T3	1 (2)	1 (3)	2 (4)	4 (3)
T4	42 (98)	34 (97)	55 (97)	131 (97)
Clinical node category (primary)				
N negative	9 (21)	12 (34)	15 (26)	36 (27)
N positive	34 (79)	23 (66)	42 (74)	99 (73)
Clinical metastasis, cM1 (primary)	7 (16)	9 (26)	8 (14)	24 (18)
Surgery type				
TPE	26 (61)	25 (71)	46 (81)	97 (72)
APE	10 (23)	8 (23)	8 (14)	26 (19)
PPE	7 (16)	2 (6)	3 (5)	12 (9)
With bony excision	1 (2)	1 (3)	4 (7)	6 (4)
With pubectomy	0 (0)	0 (0)	1 (2)	1 (1)
With sacrectomy	1 (2)	1 (3)	3 (5)	5 (4)
Urinary reconstruction				
No reconstruction	7 (16)	2 (6)	3 (5)	12 (9)
Ileal conduit urinary diversion	35 (81)	30 (86)	48 (84)	113 (84)
Others	1 (2)	3 (9)	6 (11)	10 (7)

*Note:* Data are expressed as *n* (%) or median (IQR).

Abbreviations: APE, anterior pelvic exenteration; BMI, body mass index; IQR, interquartile range; LPE, laparoscopic pelvic exenteration; OPE, open pelvic exenteration; PPE, posterior pelvic exenteration; RPE, robot‐assisted pelvic exenteration; TPE, total pelvic exenteration.

### 3.2 Clinical Characteristics of Patients Undergoing RPE

The RPE cohort comprised 43 patients. The median age was 66 years, 36 patients (84%) were men, and the median body mass index was 22 kg/m^2^. Ten patients (23%) had an ASA‐PS of 3 or higher, 23 (54%) had a Brinkman index of 400 or higher, and 13 (30%) had a history of abdominal surgery.

Neoadjuvant chemotherapy and radiotherapy were administered to 29 (67%) and 13 (30%) patients, respectively. Clinical T4 disease was present in 42 patients (98%), clinically positive lymph nodes in 34 (79%), and cM1 disease at initial diagnosis in seven (16%).

TPE, APE, and PPE were performed in 26 (61%), 10 (23%), and seven (16%) patients, respectively. One patient (2%) underwent concomitant sacrectomy; no patient underwent pubectomy. Ileal conduit urinary diversion was performed in 35 patients (81%), while seven (16%) required no urinary reconstruction.

### 3.3 Perioperative and Postoperative Outcomes

The perioperative and postoperative outcomes of the full multicenter cohort according to surgical approach are shown in Table 2. In the entire cohort, the median operative time was 688 min, and the median intraoperative blood loss was 790 mL. Intraoperative transfusion was administered to 63 patients (47%). RM0 resection was achieved in 124 patients (92%), and the median tumor size was 57 mm.

**TABLE 2:** Perioperative and postoperative outcomes.

	RPE	OPE	LPE	Total
	*N* = 43	*N* = 35	*N* = 57	*N* = 135
**Intraoperative outcomes**				
Operative time (min)	690 (565–795)	623 (529–746)	690 (567–845)	688 (567–824)
Intraoperative blood loss (mL)	360 (178–735)	2081 (964–2877)	560 (230–1405)	790 (300–1950)
Intraoperative transfusion	13 (30)	27 (77)	23 (40)	63 (47)
Intraoperative transfusion (mL)^a^	280 (280–560)	1120 (560–2040)	560 (280–940)	840 (490–900)
Resection margin status				
RMX	0 (0)	1 (3)	1 (2)	2 (1)
RM0	42 (98)	28 (80)	54 (95)	124 (92)
RM1	1 (2)	6 (17)	2 (4)	9 (7)
Tumor size (mm)	54 (40–78)	70 (48–100)	60 (50–72)	57 (40–80)
**Postoperative outcomes**				
Death within 30 days	0 (0)	0 (0)	0 (0)	0 (0)
Reoperation during hospitalization	0 (0)	4 (11)	4 (7)	8 (6)
Morbidity within 30 days				
≥ CD Grade II	16 (37)	28 (80)	38 (67)	82 (61)
Organ space SSI	5 (12)	6 (17)	17 (30)	28 (21)
Ileus	5 (12)	9 (26)	11 (19)	25 (19)
Urinary tract infection	1 (2)	4 (11)	12 (21)	17 (13)
≥ CD Grade III	7 (16)	10 (29)	18 (32)	35 (26)
Organ space SSI	3 (7)	3 (9)	9 (16)	15 (11)
Ileus	2 (5)	2 (6)	4 (7)	8 (6)
Bowel obstruction	0 (0)	0 (0)	3 (5)	3 (2)
Anastomotic leakage	2 (5)	0 (0)	0 (0)	2 (1)
Urinary tract stenosis/leakage	1 (2)	0 (0)	1 (2)	2 (1)
Duration of hospital stay (days)	23 (21–29)	34 (25–47)	34 (26–47)	31 (23–46)
Morbidity > 30 days and ≤ 90 days				
≥ CD Grade III	5 (12)	3 (9)	8 (14)	16 (12)

*Note:* Data are expressed as *n* (%) or median (IQR).

Abbreviations: CD, Clavien‐Dindo; IQR, interquartile range; LPE, laparoscopic pelvic exenteration; OPE, open pelvic exenteration; RPE, robot‐assisted pelvic exenteration; SSI, surgical site infection.

^a^ Of the patients who received transfusions.

In the RPE cohort, the median operative time was 690 min, and the median intraoperative blood loss was 360 mL. Thirteen patients (30%) received an intraoperative transfusion; among these patients, the median transfusion volume was 280 mL. Conversion to open surgery occurred in one patient (2%). The median tumor size was 54 mm. RM0 resection was achieved in 42 patients (98%), whereas RM1 resection occurred in one patient (2%).

Within 30 days after RPE, CD Grade II or higher complications occurred in 16 patients (37%). The most frequent complications were organ‐space surgical site infection and ileus, each occurring in five patients (12%), followed by urinary tract infection in one patient (2%). CD Grade III or higher complications occurred in seven patients (16%), including organ‐space surgical site infection in three (7%), ileus in two (5%), anastomotic leakage in two (5%), and urinary tract stenosis or leakage in one (2%).

No patient undergoing RPE required reoperation during hospitalization or died within 30 days. CD Grade III or higher morbidity occurring between 31 and 90 days was observed in five patients (12%). The median postoperative length of stay after RPE was 23 days.

### 3.4 Temporal Trends in Surgical Approach

The annual numbers of PE procedures according to surgical approach are shown in Figure 2. RPE was first performed in 2019, with the annual number increasing from five procedures in 2019 to six in 2020, 13 in 2021, and 19 by October 2022. RPE became the most frequently used approach in 2021 and accounted for 19 of the 26 PE procedures (73%) performed in 2022. These findings illustrate the progressive adoption of RPE during the latter part of the study period.

**FIGURE 2:**
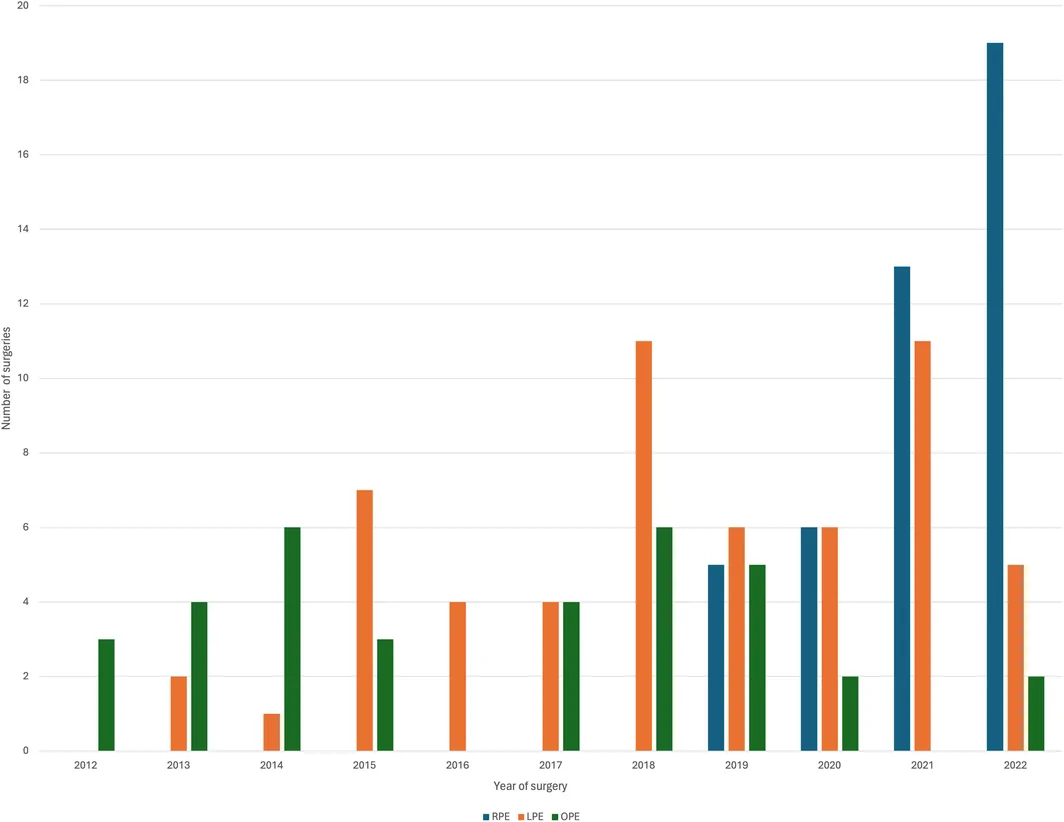
Annual trends in the number of PE procedures according to surgical approach. RPE, robot‐assisted pelvic exenteration; LPE, laparoscopic pelvic exenteration; OPE, open pelvic exenteration.

### 3.5 Supplementary Exploratory Comparative Analyses

Propensity score matching yielded 31 RPE–OPE pairs, 33 RPE–LPE pairs, and 32 minimally invasive PE–OPE pairs. The baseline characteristics and perioperative outcomes of the matched cohorts are presented in Figures S1 and S2 and Tables [Link], [Link], [Link], [Link], [Link], [Link]. These analyses were performed to provide clinical context among patients with overlapping measured characteristics and were not used as the primary basis for the study conclusions.

## 4 Discussion

In this multicenter Japanese secondary analysis, RPE for primary rectal cancer was progressively adopted and performed with acceptable perioperative outcomes in selected patients. Among 43 patients who underwent RPE, conversion to open surgery was required in one patient, RM0 resection was achieved in 98%, and no patient underwent reoperation during hospitalization or died within 30 days. CD Grade II or higher and Grade III or higher complications within 30 days occurred in 37% and 16% of patients, respectively. The main value of this study lies in describing the clinical implementation, patient characteristics, and perioperative outcomes of RPE rather than in establishing its superiority over OPE or LPE.

In the RPE cohort, clinical T4 disease was present in 98%, clinically positive lymph nodes in 79%, and cM1 disease at initial diagnosis in 16%. TPE was performed in 61% of patients, while the remaining patients underwent APE or PPE. Thus, TPE accounted for the majority of RPE procedures in this cohort, indicating that the use of RPE was not limited to APE or PPE, in which selected pelvic organs are preserved. Furthermore, 23% of the patients had an ASA‐PS of 3 or higher, showing that RPE was also performed in patients with severe systemic disease. However, only one patient underwent concomitant bony excision. Detailed information regarding pelvic sidewall fixation, vascular involvement, pelvic fibrosis, fistula or abscess formation, sacral invasion level, and reconstructive complexity was not collected uniformly. Therefore, the present data cannot define formal anatomical or technical criteria for patient selection for RPE.

The temporal trends showed a progressive increase in the use of RPE after its introduction in 2019. RPE became the most frequently used approach in the latter part of the study period, accounting for 73% of PE procedures performed through October 2022. This trend suggests that RPE was increasingly incorporated into clinical practice at the participating institutions. However, it should not be interpreted as evidence that RPE replaced OPE or LPE because changes in patient selection, robotic availability, institutional experience, and referral patterns may also have influenced the distribution of surgical approaches over time.

The implementation of RPE requires experience beyond that required for standard robot‐assisted rectal resection. PE involves multivisceral resection and may require urinary diversion, complex reconstruction, and management of major intraoperative complications. In the present RPE cohort, 81% of patients underwent ileal conduit urinary diversion. Safe implementation is therefore likely to depend not only on the experience of the operating surgeon but also on sufficient institutional experience with PE and an established perioperative care system. Because surgeon‐level procedural volume, training pathways, and institutional criteria for introducing RPE were not collected, the present study cannot define a minimum case volume or standardized implementation pathway.

The perioperative outcomes observed in the RPE cohort support its feasibility in selected patients. The median operative time was 690 min, highlighting the complexity and prolonged operative time associated with the procedure. The median intraoperative blood loss was 360 mL, and 30% of patients received an intraoperative transfusion. Within 30 days, CD Grade II or higher complications occurred in 37% of patients, whereas CD Grade III or higher complications occurred in 16%. No patient required reoperation during hospitalization, and no 30‐day mortality occurred. The median postoperative length of stay was 23 days. Although PE remains a highly invasive procedure with substantial morbidity, these findings indicate that RPE could be completed with acceptable short‐term outcomes in the patients selected for this approach.

The robotic platform may provide technical advantages during PE through three‐dimensional magnified visualization, articulated instruments, tremor filtration, motion scaling, and a stable operative field. These features may facilitate precise dissection and hemostasis in the confined pelvis. Previous studies have also reported lower blood loss with minimally invasive PE than with OPE [4]. Perioperative transfusion has been associated with postoperative morbidity and worse long‐term outcomes after colorectal surgery [21], providing a clinical rationale for minimizing intraoperative blood loss. However, the outcomes observed in the present study cannot be attributed solely to the robot‐assisted approach. Tumor extent, invasion pattern, previous treatment, anticipated resection planes, reconstruction requirements, institutional experience, and year of surgery may have influenced both the selection of surgical approach and the observed outcomes.

The learning curve should also be considered when interpreting the implementation of RPE. Previous studies have suggested that approximately 30 procedures may be required to overcome the learning curve for robot‐assisted colorectal surgery [22, 23]. However, PE is substantially more heterogeneous and complex than standard rectal resection, and experience with conventional robot‐assisted colorectal surgery may not directly translate into proficiency in RPE. The learning process is likely to include multivisceral dissection, urinary and intestinal reconstruction, intraoperative decision‐making, coordination among the involved departments, and an appropriate conversion strategy. RPE should therefore be introduced cautiously at institutions with sufficient experience in both robot‐assisted surgery and PE.

RM0 resection was achieved in 42 of the 43 patients undergoing RPE. Although this finding is clinically encouraging, it should be interpreted cautiously. Resection margin status after PE is strongly influenced by tumor extent, invasion pattern, fixation to adjacent structures, and the selection of patients considered technically suitable for a minimally invasive approach. Moreover, anatomical factors directly affecting resectability were not uniformly collected. Consequently, the high RM0 rate should be interpreted as showing that oncologically appropriate resection was achievable in selected patients, rather than as evidence of the oncological superiority of RPE.

RPE should therefore be considered a complementary surgical approach rather than a replacement for OPE. RPE may be considered when R0 resection is judged technically feasible using a robot‐assisted approach and when the anticipated extent of multivisceral resection and reconstruction can be safely managed at an institution with sufficient experience in PE. In contrast, OPE remains essential when wide exposure, rapid vascular control, extensive pelvic sidewall or bony resection, or complex reconstruction is required. Severe pelvic inflammation, fibrosis, abscess or fistula formation, and major vascular involvement may also favor an open approach. Because these factors were not systematically collected in this study, they should be regarded as general clinical considerations rather than selection criteria derived from the present cohort. The operative approach should be individualized according to tumor anatomy, technical feasibility, and institutional experience.

The propensity score–matched analyses were retained as supplementary exploratory analyses to provide clinical context among patients with overlapping measured characteristics. Although these analyses suggested favorable perioperative profiles for RPE in selected matched cohorts, they cannot adequately account for confounding by indication. The propensity score model did not include several important determinants of operative complexity, including pelvic sidewall fixation, fibrosis, inflammation, fistula formation, vascular involvement, sacral invasion level, and reconstructive complexity. Therefore, the matched analyses should be regarded as hypothesis‐generating and were not used as the primary basis for the study conclusions.

This study has several limitations. First, its retrospective design and the absence of standardized criteria for selecting the surgical approach introduced selection bias and confounding by indication. Second, important anatomical and technical determinants of surgical complexity were not uniformly available. Third, all participating institutions had performed at least one RPE for a pelvic malignant tumor, which may limit the generalizability of the findings to institutions without experience in RPE. Fourth, the study period extended from 2012 to 2022 and encompassed changes in robotic technology, perioperative management, institutional experience, and patient selection. Fifth, the RPE cohort included only 43 patients, and surgeon‐ and institution‐level learning curves could not be evaluated. Sixth, bony excision was performed in only six patients in the entire cohort and in only one RPE patient, limiting the applicability of the findings to the most technically demanding PE procedures. Finally, the supplementary propensity score–matched analyses adjusted only for measured variables and cannot establish comparative effectiveness among surgical approaches.

In conclusion, RPE was progressively adopted and performed with acceptable perioperative outcomes in selected patients undergoing PE for primary rectal cancer at Japanese institutions. RPE may serve as a complementary surgical approach at institutions with sufficient experience in PE, whereas OPE remains essential for patients with extensive or technically demanding disease. Further studies are needed to define reproducible criteria for patient selection, requirements for safe implementation, and long‐term oncological outcomes.

## Author Contributions

**Shoma Yamamoto:** conceptualization, investigation, writing – original draft, methodology, visualization, data curation. **Hiroyasu Kagawa:** supervision, writing – review and editing, conceptualization, project administration. **Yusuke Yatabe:** supervision, investigation. **Yusuke Kinugasa:** supervision, conceptualization.

## Funding

The authors have nothing to report.

## Ethics Statement

This study was conducted in accordance with the ethical standards of the institutional research committee and with the Declaration of Helsinki (64th WMA General Assembly, Fortaleza, Brazil, October 2013). All data were anonymized before analysis. This study was approved by the Tokyo Medical and Dental University's Ethics Review Committee (M2022‐275) and the Tokushukai Group Ethics Committee (TGE02189‐025) for all facilities.

## Consent

Data for this study were collected retrospectively from existing medical records created during routine clinical practice. Written informed consent had been obtained from each patient for surgical and clinical procedures.

## Conflicts of Interest

Y.K. is an Editorial Board Member of Annals of Gastroenterological Surgery and has received honoraria from Johnson & Johnson K.K., Intuitive Surgical G.K., and Medtronic Japan Co. Ltd., as well as research funding from Medicaroid Co. Ltd. H.K. has received honoraria from Johnson & Johnson K.K. and Intuitive Surgical G.K. The remaining authors declare no conflicts of interest related to this study.

## Supporting information


**Figure S1:** Flow diagram of the propensity score–matched analyses comparing RPE with OPE and RPE with LPE. RPE, robot‐assisted pelvic exenteration; LPE, laparoscopic pelvic exenteration; OPE, open pelvic exenteration.

**Figure S2:** Flow diagram of the propensity score–matched analysis comparing MIS‐PE with OPE. MIS‐PE, minimally invasive pelvic exenteration; OPE, open pelvic exenteration.

**Table S1:** Baseline characteristics after propensity score matching (RPE and OPE).

**Table S2:** Baseline characteristics after propensity score matching (RPE and LPE).

**Table S3:** Baseline characteristics after propensity score matching (MIS‐PE and OPE).

**Table S4:** Perioperative and postoperative outcomes after matching (RPE vs. OPE).

**Table S5:** Perioperative and postoperative outcomes after matching (RPE vs. LPE).

**Table S6:** Perioperative and postoperative outcomes after matching (MIS‐PE vs. OPE).

## Data Availability

The data that support the findings of this study are available on request from the corresponding author. The data are not publicly available due to privacy or ethical restrictions.

## Participating Institutions and Representatives

Yusuke Kinugasa^1^, Toshiki Mukai^2^, Kei Kimura^3^, Koji Yamanoi^4^, Jin Kono^5^, Mitsuru Yokota^6^, Hiroki Takahashi^7^, Akihiro Kobayashi^8^, Kenji Kobayashi^9^, Nobuki Ichikawa^10^, Masayoshi Yasui^11^, Keita Nakane^12^, Manabu Yamamoto^13^, Atsushi Takenaka^14^, Yuya Nakamura^15^, Ichiro Takemasa^16^, Norimitsu Yabusaki^17^, Shintaro Akamoto^18^, Shuichi Tatarano^19^, Kohei Murata^20^, Tatsuya Manabe^21^, Tetsuya Fujimura^22^, Mikio Kawamura^23^, Hiroyuki Egi^24^, Shigeki Yamaguchi^25^, Yoshito Terai^26^, Shigetaka Inoue^27^, Akihiro Ito^28^.

^1^Department of Gastrointestinal Surgery, Institute of Science Tokyo, 1–5‐45, Yushima, Bunkyo‐ku, Tokyo, Japan.

^2^Department of Gastroenterological Surgery, Cancer Institute Hospital, Japanese Foundation for Cancer Research, Tokyo, Japan.

^3^Division of Lower Gastrointestinal Surgery, Department of Gastroenterological Surgery, Hyogo Medical University, Hyogo, Japan.

^4^Department of Gynecology and Obstetrics, Graduate School of Medicine, Kyoto University, Kyoto, Japan.

^5^Department of Urology, Graduate School of Medicine, Kyoto University, Kyoto, Japan.

^6^Department of General Surgery, Kurashiki Central Hospital, Okayama, Japan.

^7^Department of Gastroenterological Surgery, Nagoya City University Graduate School of Medical Sciences, Nagoya, Japan.

^8^Department of Surgery, Chiba‐Nishi General Hospital, Chiba, Japan.

^9^Department of Gastroenterological Surgery, Kariya Toyota General Hospital, Aichi, Japan.

^10^Department of Gastroenterological Surgery I, Hokkaido University Graduate School of Medicine, Hokkaido, Japan.

^11^Department of Gastroenterological Surgery, Osaka International Cancer Institute, Osaka, Japan.

^12^Department of Urology, Graduate School of Medicine, Gifu University, Gifu, Japan.

^13^Division of Gastrointestinal and Pediatric Surgery, Department of Surgery, Faculty of Medicine, Tottori University, Yonago, Japan.

^14^Division of Urology, Department of Surgery, Faculty of Medicine, Tottori University, Yonago, Japan.

^15^Department of Surgery, National Hospital Organization, Himeji Medical Center, Hyogo, Japan.

^16^Department of Surgery, Surgical Oncology and Science, Sapporo Medical University, Hokkaido, Japan.

^17^Department of Surgery, Okazaki City Hospital, Aichi, Japan.

^18^Department of Surgery, Sumitomo Besshi Hospital, Ehime, Japan.

^19^Department of Urology, Kagoshima University Graduate School of Medical and Dental Sciences, Kagoshima, Japan.

^20^Department of Surgery, Kansai Rosai Hospital, Hyogo, Japan.

^21^Department of Surgery, Faculty of Medicine, Saga University, Saga, Japan.

^22^Department of Urology, Jichi Medical University, Tochigi, Japan.

^23^Department of Gastrointestinal and Pediatric Surgery, Institute of Life Sciences, Mie University Graduate School of Medicine, Mie, Japan.

^24^Department of Gastrointestinal Surgery and Surgical Oncology, Ehime University Hospital, Ehime, Japan.

^25^Division of Colorectal Surgery, Department of Surgery, Tokyo Women's Medical University, Tokyo, Japan.

^26^Department of Obstetrics and Gynecology, Kobe University Graduate School of Medicine, Hyogo, Japan.

^27^Department of Surgery, Fukuoka Red Cross Hospital, Fukuoka, Japan.

^28^Department of Urology, Tohoku University Graduate School of Medicine, Miyagi, Japan.
